# Bilateral Superficial Cervical Plexus Block to Avoid Airway Manipulation in a Patient With Tracheal Compression and Bleeding Nodular Tracheal Infiltration: A Safe and Feasible Option

**DOI:** 10.7759/cureus.71693

**Published:** 2024-10-17

**Authors:** Aruna Parameswari, Kishore Manivannan, Jabeena Salim, Mahalakshmi Sankar, Lakshmipriya Ramu

**Affiliations:** 1 Anesthesiology, Sri Ramachandra Institute of Higher Education and Research, Chennai, IND

**Keywords:** cervical lymph node biopsy, dexmedetomidine, nodular tracheal infiltration, superficial cervical plexus block, tracheal compression

## Abstract

This case report describes the successful application of bilateral superficial cervical plexus block (SCPB) to avoid airway manipulation in a patient with tracheal compression and bleeding nodular tracheal infiltration. Our patient was a 65-year-old male with Type 2 diabetes mellitus and systemic hypertension who presented with swelling in the anterior aspect of the right neck, difficulty swallowing, and hemoptysis. Imaging revealed significant tracheal compression and nodular tracheal infiltration that was bleeding on touch. Due to the complex tumor infiltration of the trachea, SCPB was chosen over general anesthesia. The procedure involved unilateral SCPB with intravenous dexmedetomidine initially, followed by SCPB on the other side also to effectively manage change in surgical procedure intraoperatively. This case report emphasizes the potential of SCPB with intravenous dexmedetomidine as an alternative to general anesthesia (GA), especially in patients with difficult airways due to complex tumor infiltration of the trachea.

## Introduction

Traditionally, patients undergoing procedures around the neck have been given general anesthesia (GA) to facilitate surgical intervention. However, recent advancements in anesthetic techniques have introduced a novel alternative: the superficial cervical plexus block (SCPB) for procedures in the anterior and anterolateral aspects of the neck, providing both analgesia and anesthesia [1].

This case report deals with the application of SCPB with intravenous dexmedetomidine in a patient with extrinsic tracheal compression and nodular tracheal infiltration posted for a diagnostic cervical lymph node biopsy. By exploring the efficacy and safety of SCPB in this context, we highlight its role as a valuable alternative to GA, particularly in scenarios where airway manipulation carries heightened risks.

## Case presentation

Patient information and clinical findings

A 65-year-old male weighing 70 kg with Type 2 diabetes mellitus and systemic hypertension on regular medications presented with swelling in the right anterior neck for the past three years. The patient reported an increase in the size of the swelling over the past two months, accompanied by difficulty in swallowing and episodes of hemoptysis. He denied any history of difficulty breathing, noisy breathing, or difficulty lying supine. An airway examination revealed a Mallampati class II score with adequate mouth opening and normal neck movements. A systemic examination and all blood investigations were within normal limits.

Diagnostic assessment

**Figure 1:**
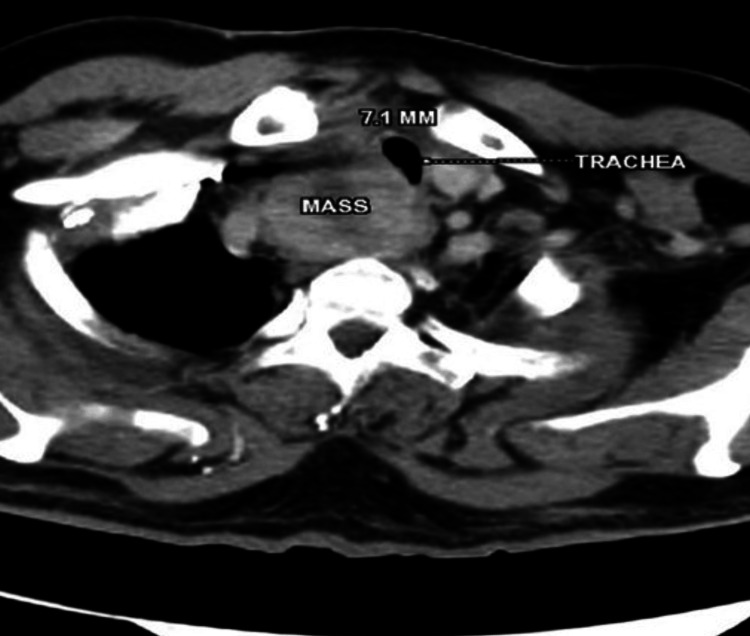
Transverse section of PET-CT at C7-D1 level. Transverse section of PET-CT showing lesion in right paraesophageal groove at C7-D1 level with compression of the trachea and narrowest lumen diameter of 7.1 mm.

**Figure 2:**
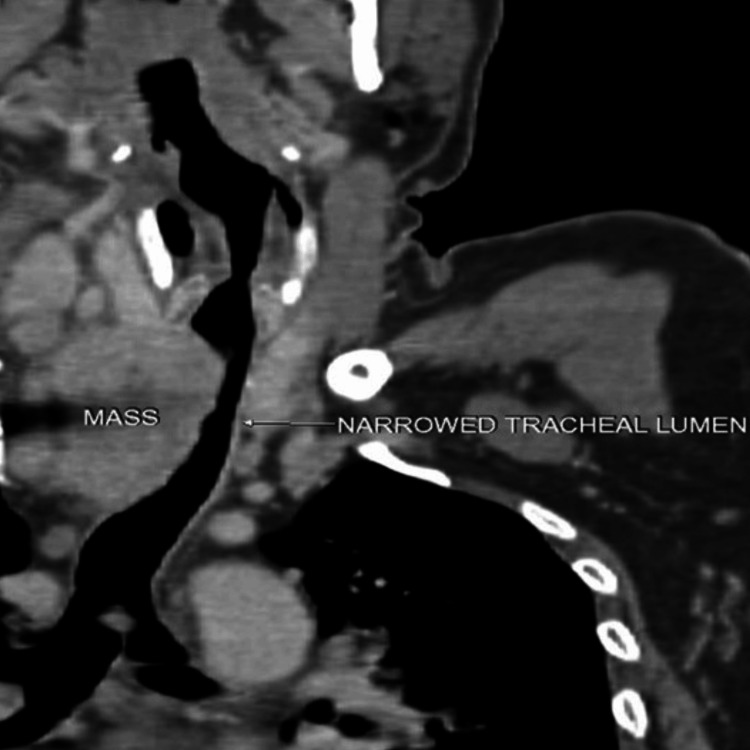
Sagittal section of PET-CT showing narrowed tracheal lumen at C7-D1 vertebral level.

**Figure 3:**
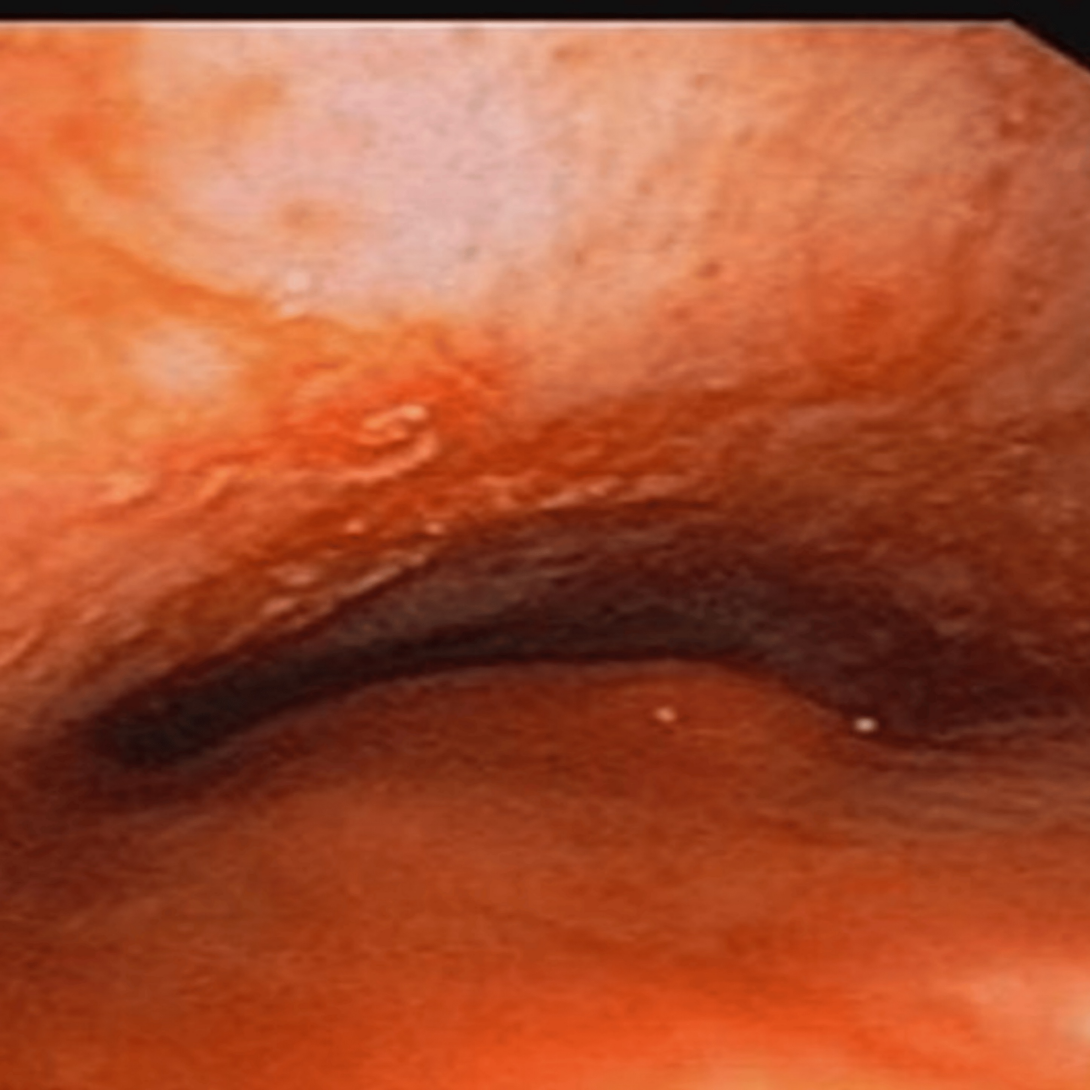
Flexible video bronchoscopy showing tracheal lumen narrowing with nodular infiltration.

PET-CT revealed a mass measuring 55x49x54 mm in the right paraesophageal area, compressing the trachea and encasing the right common carotid artery, with a level four lymph node in the right side of the neck measuring 22x17 mm causing severe compression of the internal jugular vein as shown in Figure 1 and Figure 2. Multiple metastases were present in the bones, lungs, and mediastinum. Flexible video bronchoscopy revealed right vocal cord palsy with narrow glottic chink, subglottic tracheal compression, and nodular mucosal infiltration of the trachea, which bled on touch (Figure 3).

Surgeons opted to perform a biopsy from the level four cervical lymph node on the right side of the neck, which could help them plan the further course of the patient’s treatment. Considering the complexity of airway management in this case, due to complex tumor infiltration, a USG-guided right-sided superficial cervical plexus block along with IV dexmedetomidine sedation was planned.

Therapeutic intervention

Written informed consent was obtained. Oral hypoglycaemic agents were withheld, antihypertensives were continued on the day of surgery, and the patient was shifted to the operating room. All American Society of Anesthesiologists (ASA)-standard monitors were attached and baseline vitals were noted: heart Rate - 83/minute, blood Pressure - 130/90 mmHg, and Spo2 - 98% RA. Intravenous dexmedetomidine 1 mcg/kg was given over 10-15 minutes, followed by continuous infusion at the rate of 0.5-1 mcg/kg/hour. The patient was positioned supine with his head turned to the left side. Under sterile aseptic precautions, a right-sided SCPB was administered with 10 ml of 0.5% isobaric bupivacaine under USG guidance with the help of a high-frequency linear probe.

Sensory loss was confirmed using ice packs before the surgery commenced. Since the nodal mass was in close proximity to the internal jugular vein, the surgeon faced significant difficulty in obtaining an excision biopsy. Given the challenging nature of the mass location and its relation to critical vascular structures, it was decided to perform a biopsy from the thyroid nodule through a midline incision in the neck. To facilitate this, a USG-guided left SCPB with 10 ml of 0.5% isobaric bupivacaine was administered. The procedure proceeded uneventfully. The patient was shifted to the post-anesthesia care unit (PACU) with stable vitals and subsequently to the ward after four hours.

## Discussion

The superficial cervical plexus (SCP) originates from the C1-C4 spinal nerves and gives rise to four cutaneous branches: the greater auricular, lesser occipital, transverse cervical, and supraclavicular nerves. SCPB involves injecting local anesthetic subcutaneously at the posterior margin of the sternocleidomastoid muscle, targeting the cutaneous branches of the cervical plexus [2]. 

Wilson et al. performed a systematic review and meta-analysis evaluating the efficacy of bilateral SCPB (BSCPB) in patients undergoing thyroid surgery, and they found a significant reduction in opioid consumption and lower visual analog scale (VAS) scores at 24 hours postoperatively [3]. Numerous studies have been published in the literature quoting the efficacy of SCPB for procedures like carotid endarterectomies and cervical lymph node biopsies [4]. In one such case report by Ahuja et al., SCPB was given for a cervical lymph node biopsy in a high-risk patient with coronary artery disease (CAD) and chronic obstructive pulmonary disease (COPD), which reduced perioperative cardiopulmonary complications [5].

Our case report highlights the successful application of BSCPB to avoid airway manipulation in a patient with tracheal compression and bleeding nodular tracheal infiltration undergoing a cervical lymph node biopsy.

The decision to opt for SCPB over GA initially was primarily because of the challenges posed by extrinsic tracheal compression and nodular infiltration of the trachea, which was bleeding on touch. With only SCPB and mild sedation, we were able to provide adequate analgesia while at the same time, reducing the risk of airway manipulation with GA. 

The intraoperative challenges demanded swift adaptation during the procedure. The options included converting to GA with manipulation of the airway or providing anesthesia to the surgical site by blocking the SCP on the other side. To avoid the risks associated with airway manipulation in a patient with tracheal compression and nodular tracheal infiltration that was bleeding on touch, SCPB of the other side was given and the procedure was successfully completed. We were able to provide adequate analgesia without unwanted airway manipulation that could have compromised patient safety due to the complexity of the intraoperative situation. Given the risks associated with airway manipulation, a backup plan for airway management was in place. We had prepared for two alternative strategies: (a) awake fiberoptic intubation with the help of a 3.5 mm fibreoptic scope, which was kept ready in the airway cart, and (b) tracheostomy under local anesthesia. Fortunately, these interventions were not required, as BSCPB provided sufficient analgesia. 

However, it is essential to note that while SCPB can be an effective alternative to GA in cases involving superficial neck procedures, it may not be suitable for deeper surgeries in the neck, such as those involving deeper cervical spaces.

## Conclusions

This case demonstrates the successful use of BSCPB combined with intravenous dexmedetomidine sedation as an alternative to GA for a patient with significant airway risks. The decision to avoid airway manipulation, particularly in this patient with extrinsic tracheal compression and bleeding nodular tracheal infiltration, was critical in minimizing the potential complications associated with GA.

By utilizing SCPB, we achieved sufficient analgesia while ensuring patient safety, avoiding the need for intubation or airway instrumentation. This approach may be especially valuable in high-risk cases where airway compromise is a concern. Further studies may help to solidify the role of SCPB in similar complex cases, offering a safer alternative for patients with compromised airways
